# Correlation of biomarkers thiobarbituric acid reactive substance, nitric oxide and central subfield and cube average thickness in diabetic retinopathy: a cross-sectional study

**DOI:** 10.1186/s40942-016-0033-z

**Published:** 2016-03-08

**Authors:** Surabhi Ruia, Sandeep Saxena, S. Prasad, Shashi R. Sharma, Levent Akduman, Vinay K. Khanna

**Affiliations:** 1 Department of Ophthalmology, King George’s Medical University, Lucknow, India; 2 Department of Community Medicine, King George’s Medical University, Lucknow, India; 3 Department of Ophthalmology, Saint Louis University Eye Institute, Saint Louis University, Saint Louis, MO USA; 4 Developmental Toxicology Division, Indian Institute of Toxicology Research, Lucknow, India

**Keywords:** Thiobarbituric acid reactive substance, Nitric oxide, Oxidative stress, Diabetic retinopathy, Spectral domain optical coherence tomography, Central subfield thickness, Cube average thickness

## Abstract

**Background:**

To evaluate the role of thiobarbituric acid reactive substance (TBARS) and nitric oxide (NO) as biochemical biomarkers and central subfield (CST) and cube average thickness (CAT) as biomarkers for medical imaging in diabetic retinopathy.

**Methods:**

Forty consecutive cases of diabetic retinopathy and 20 healthy controls were included. Cases were divided into two groups: non proliferative diabetic retinopathy (n = 20) and proliferative diabetic retinopathy (n = 20) according to ETDRS classification. LogMAR visual acuity was documented. Plasma levels of TBARS, NO and glycated hemoglobin (HbA1c) were measured using standard protocol. CST and CAT were analyzed on spectral domain optical coherence tomography. Data was analyzed statistically.

**Results:**

Increased severity of diabetic retinopathy was associated with an increase in plasma levels of TBARS (F = 10.92; p < 0.001), NO (F = 21.8; p < 0.001) and HbA1c (F = 5.87; p = 0.001). Increase in CST (F = 61.51; p < 0.001) and CAT (F = 60.84; p < 0.001) was also found to be associated with increased severity of diabetic retinopathy. Pearson’s correlation analysis revealed a positive correlation of TBARS with CST (r = 0.29; p = 0.038) and CAT (r = 0.31; p = 0.04). A positive correlation of NO with CST (r = 0.27; p = 0.03) and CAT (r = 0.7; p = 0.001) was also observed. On univariate analysis with logMAR visual acuity as dependent variable, a significant increase in visual acuity was observed with increase in independent variables TBARS (B = 0.22; p = 0.004), NO (B = 0.006; p < 0.001), CST (B = 0.005; p < 0.001) and CAT (B = 0.005; p < 0.001). On multivariate linear regression analysis with logMAR visual acuity as dependent variable and adjusting for other factors like duration of diabetes and HbA1c, it was observed that increase in independent variables TBARS (B = 0.07), NO (B = 0.001) and CST (B = 0.004) independently predict increase in logMAR visual acuity (p < 0.001).

**Conclusion:**

Thiobarbituric acid reactive substance and nitric oxide serve as potential biochemical markers whereas central subfield and cube average thickness serve as potential biomarkers for medical imaging for severity of diabetic retinopathy. In a clinical retinal setting, CAT and CST will help in early recognition of increase in severity of diabetic retinopathy.

## Background

Diabetic retinopathy is the most frequent cause of blindness among adults aged 20–74 years [1]. The number of people with diabetes is expected to rise to 300 million worldwide by 2025 [2]. Diabetic macular edema resulting from blood retinal barrier breakdown (BRB) in diabetic retinopathy, is the most common cause of qualitative as well as quantitative reduction in health-related quality of life [3].

The pathophysiology of DR is complex and theories as to the mechanisms involved come from cell culture experiments and animal models. From experimental data, an understanding of what might happen in humans is proposed. The high content of polyunsaturated fatty acids, high oxygen uptake and glucose oxidation relative to any other tissue, renders the retina more susceptible to oxidative stress [4]. Increased concentration of reactive oxygen species (ROS) is considered as a causal link between hyperglycemia and diabetic complication through various metabolic pathways. These include the polyol pathway [5], the advanced glycation end product (AGE) pathway [6], protein kinase C (PKC) pathway [7, 8] alteration in the expressions of vascular endothelial growth factor (VEGF) [9] and mitochondrial dysfunctions [10]. These pathways trigger further production of ROS and subsequent lipid peroxidation, resulting in amplified tissue damage [11].

Irreversible accumulation of AGE occurs within retinal capillary cells in the later stages of retinopathy. AGE lead to the activation of nuclear transcriptional factor, NF-kB, by generating ROS [12]. NF-kB activation triggers a pro-apoptotic program in retinal pericytes [13]. ROS mediated activation of PKC leads to increasing vessel permeability, endothelial proliferation and apoptosis, via upregulation of angiogenic factor VEGF [14]. Inhibition of PKC activation is shown to prevent diabetes-induced oxidative stress [15, 16]. Altered gene profile of scavenging enzymes in retinal pericytes obtained from diabetic patients, correlates with the over expression of the cell death protease gene, suggesting an important role of oxidative stress in pericyte loss seen in diabetic retinopathy [17]. Damage to the mitochondrial lipid membrane by ROS increases the permeability of the organelle, resulting in increased apoptosis of retinal capillary cells [12].

Hyperglycemia induced expression of nitric oxide synthase (eNOS) and superoxide anions (O_2_^−^) by endothelial cells has been observed in vitro [18]. AGE initiate a sequence of events leading to retinal capillary cell apoptosis via activation of NF-kB [19]. NF-kB modulates the expression of inducible nitric oxide synthase (iNOS), resulting in increased ROS production [20]. Nitric oxide (NO) is instrumental in vasodilation of retinal vessels in diabetic retinopathy. Reaction between superoxide (ROS) and NO forms peroxynitrite (ONOO^−^) and reduces the bioavailability of NO. Elevated peroxynitrite levels in circulation have been documented in diabetics and is known to cause endothelial dysfunction [21]. Caspases, a group of cysteine proteases that are essential for mediating apoptosis in cells [22], are known to be very sensitive toward oxidative and nitrative stress [23]. Caspase-3 is activated in the retina in diabetes, and the therapy that inhibits the development of retinopathy in diabetic rats also inhibits retinal caspase-3 activation [24].

Reliable, reproducible quantitative estimation of diabetic macular edema has been realized with advent of spectral domain optical coherence tomography (SD-OCT). Macular thickness parameters on SD-OCT have been well correlated with severity of diabetic retinopathy [25]. Biomarkers are defined as anatomical, biochemical, molecular parameters or imaging features used for refinement of diagnosis, measuring progress of disease or predict or monitor effects of treatment. They are measured by laboratory assay, physical examination or medical imaging. Their source can be tissue or body fluid such as serum/plasma, urine, synovial fluid or tissue biopsy [26]. Imaging biomarkers have the benefit of targeting the disease focus in comparison to biochemical biomarkers which tend to integrate information from entire body. Earlier studies have documented the possible role of oxidative and nitrative stress markers, and role of OCT in identifying subclinical macular edema as biomarkers in diabetic retinopathy [27]. However, these studies have not correlated the biomarkers in serum with biomarkers of medical imaging.

The purpose of our study was to correlate the serum biomarkers, TBARS and NO with biomarkers for medical imaging, CAT and CST. The objectives of our study were: (1) to assess the potential role of thiobarbituric acid reactive substance (TBARS) and nitric oxide (NO) as biochemical biomarkers whereas central subfield (CST) and cube average thickness (CAT) as biomarkers for medical imaging for diabetic retinopathy, (2) to identify the role of these biomarkers as predictors of visual impairment in diabetic retinopathy.

## Methods

Our study had institutional review board clearance from ethics committee of King George Medical university, Lucknow, Uttar Pradesh, India. The study was performed in accordance to the tenets of the Helsinki declaration. Tertiary care center based prospective cross sectional study, where 40 consecutive cases and 20 healthy controls were included after obtaining informed voluntary consent. These 40 cases were divided into non proliferative diabetic retinopathy (NPDR; n = 20) and proliferative diabetic retinopathy (PDR; n = 20) on the basis of Early treatment diabetic retinopathy study (ETDRS) classification [28]. Non diabetic individuals presenting for refraction were included as controls. Cases with ocular diseases which could affect the retinal vascular pathology (hypertensive retinopathy), any previous ophthalmic surgical or laser interventions, fluorescein angiography suggestive of ischemic maculopathy, end stage renal disease, cases with signal strength 5 or below on OCT examination and cases taking any mineral supplements or antioxidants were excluded from the study. Best-corrected visual acuity was documented in logMAR scale. Slit lamp biomicroscopic and dilated ophthalmoscopic examination was performed. Digital fundus photography and fluorescein angiography were performed using Zeiss fundus camera FF 450 Plus with pixel width of 0.0054 and image size 2588 × 1958 (Carl Zeiss Meditec AG, Jena, Germany).

Blood samples from study subjects were drawn by aseptic vein puncture and transferred into tubes containing 3.89 % trisodium citrate (in the ratio of 9:1) for separation of plasma. The parameters for measure of oxidative stress were TBARS and NO. Glycated hemoglobin (HbA1c) was measured on autoanalyser using standard protocol.

*Assay of lipid peroxidation* As a measure of lipid peroxidation, malonaldehyde formation was estimated using the level of thiobarbituric acid reactive substances following the method of Ohkawa et al. [29]. The results are expressed as nmMDA/ml. Thiobarbituric acid was procured from Sigma-Aldrich, St. Louis, Missouri, USA. Other chemicals required such as ethylene diamine tetra acetic acid and trichloroacetic acid were procured from Merck, Mumbai, India.

*Assay of nitric oxide* Levels of NO in plasma were determined using the nitric oxide ELISA kit available commercially (Calbiochem, San Diego, USA) [30]. The results are expressed as µmoles/liter.

**Fig. 1:**
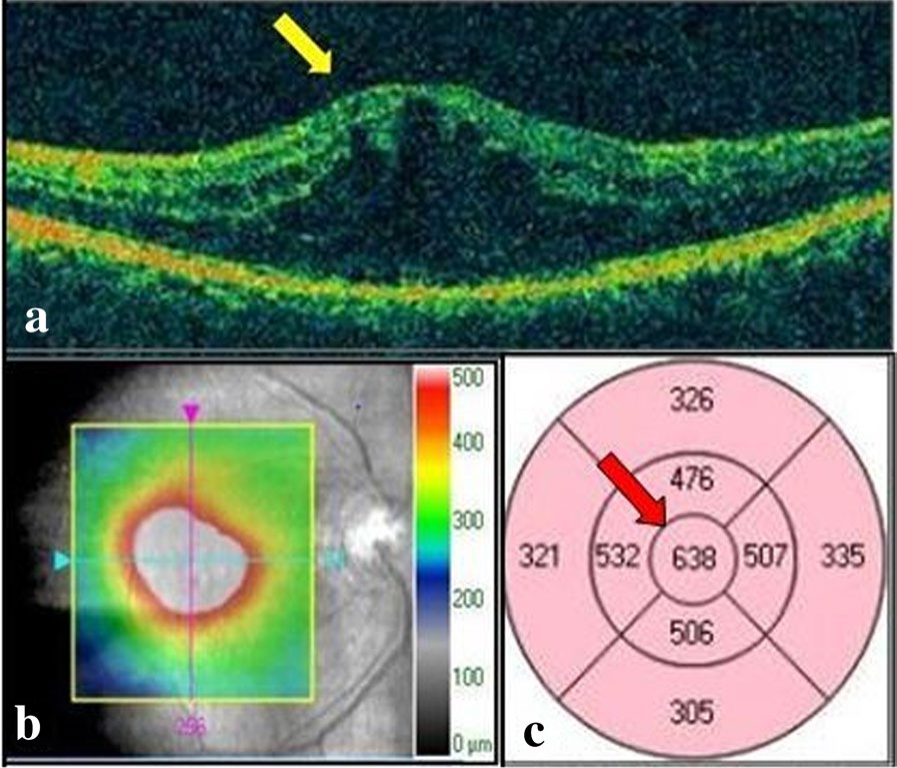
**a** Horizontal cross section scan of spectral domain optical coherence tomography illustrating diabetic macular edema (*yellow arrow*). **b** *Color* coded internal limiting membrane-retinal pigment epithelium thickness overlay map of 6X6 square mm macular cube. **c** Early treatment diabetic retinopathy study (ETDRS) grid map with numerical data for central subfield retinal thickness within innermost *circle* (*red arrow*) as well as average macular thickness

*Macular thickness measurements* Every study subject underwent imaging of the fundus using macular cube 512x128 feature of Cirrus High Definition SD-OCT (Carl Zeiss Meditec Inc., CA, USA). CST and CAT were documented in µm. CST was defined as thickness of the central circle in the circular map known as the ETDRS Grid. CAT was defined as an overall average thickness for the internal limiting membrane-retinal pigment epithelium tissue layer over the entire 6 × 6 mm square scanned area (Fig. 1).

### Statistical analysis

Data is summarized and presented as Mean ± SE. Chi square (χ^2^) test analyzed the difference in gender distribution between the groups. One way analysis of variance (ANOVA) was done to compare the values of TBARS, NO, CST, CAT and HbA1c between the study groups control, NPDR and PDR. The mean values were found to be significantly different between the groups, hence post hoc test (Fisher’s least significant difference) were done to find the difference between control and NPDR, control and PDR, NPDR and PDR. Pearson’s correlation coefficient (r) was found to analyze the correlation of the continuous variables TBARS, NO with CAT and CST. Univariate followed by multivariate linear regression analysis was done taking visual acuity as a dependent variable with TBARS, NO, CST and CAT as independent variables, and adjusted for duration of diabetes and HbA1c. p < 0.05 was considered statistically significant. All analyses were performed using SPSS software (window version 21.0).

## Results

The mean age was 54 ± 1.48 years in controls, 53.5 ± 2.68 years in NPDR and 56.2 ± 4.12 years in PDR group. No statistically significant difference existed between the cases and controls (F = 0.41; p > 0.05). The male and female ratio in different groups was 13 males and 7 females in controls, 12 males and 8 females in NPDR and 10 males and 10 females in PDR. No statistically significant difference existed in gender distribution between the cases and controls (χ^2^ = 0.42; p > 0.05). The mean duration of diabetes (years) in NPDR group was 8.2 ± 2.3 and in PDR group 11.4 ± 4.1. Mean logMAR visual acuity, CST, CAT and plasma levels of TBARS, NO, HbA1c among the study groups are summarized in Table 1.

**Table 1:** Summary of Mean ± SD of study variables among study groups along with correlation on ANOVA

Variable	Groups			F value + (3,76 DF)	p value
	Controls (n = 20)	NPDR (n = 20)	PDR (20)		
LogMAR Visual acuity	0.08 ± 0.02	0.75 ± 0.07	1.29 ± 0.07	84.72	<0.001
Central subfield thickness (µm)	249.30 ± 5.22	321.45 ± 24.25	319.03 ± 18.64	60.84	<0.001
Cube average thickness (µm)	258.93 ± 2.45	334.90 ± 10.47	335.45 ± 7.46	61.5	<0.001
TBARS (nmMDA/ml)	1.98 ± 0.15	3.11 ± 0.22	3.01 ± 0.23	10.92	<0.001
NO (µmoles/liter)	62.46 ± 13.09	109.72 ± 13.27	154.25 ± 11.77	21.8	<0.001
Glycated hemoglobin (% of total hemoglobin)	5.8 ± 0.78	7.6 ± 0.67	8.3 ± 1.1	5.87	0.001

**Fig. 2:**
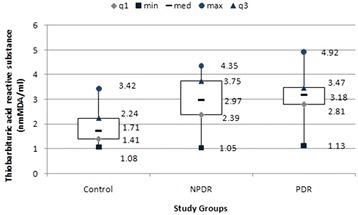
*Box* and *whisker plot* illustrating plasma levels of thiobarbituric acid reactive substances among study groups. *NPDR* non proliferative diabetic retinopathy, *PDR* proliferative diabetic retinopathy

**Fig. 3:**
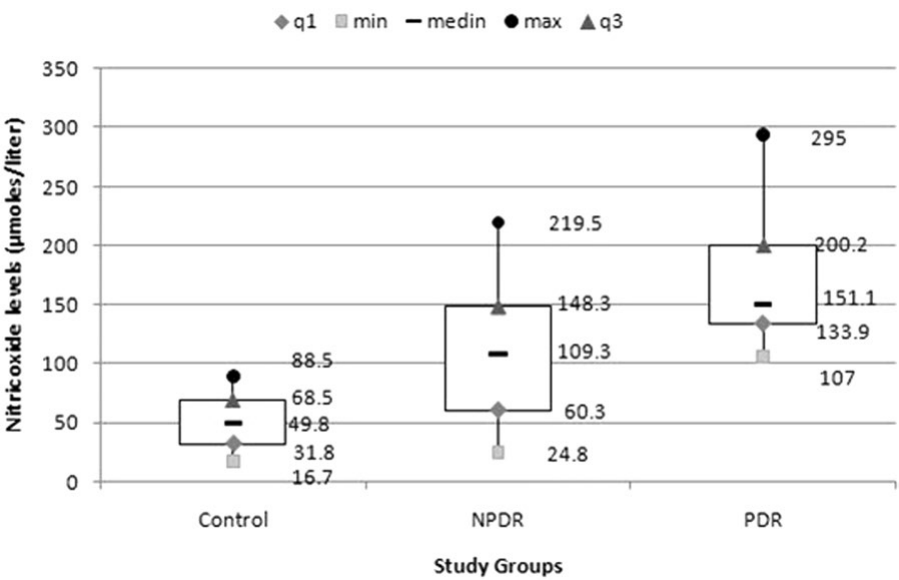
*Box* and *whisker plot* illustrating plasma levels of nitric oxide among study groups. *NPDR* non proliferative diabetic retinopathy, *PDR* proliferative diabetic retinopathy

ANOVA revealed significant increase in levels of TBARS (F = 10.92; p < 0.001), NO (F = 21.8; p < 0.001) and HbA1c (F = 5.87; p = 0.001) with increase in severity of diabetic retinopathy (Figs. 2, 3). LogMAR visual acuity (F = 84.72; p < 0.001), CAT (F = 60.84; p < 0.001) and CST (F = 61.51; p < 0.001) were also found to increase with severity of diabetic retinopathy.

**Fig. 4:**
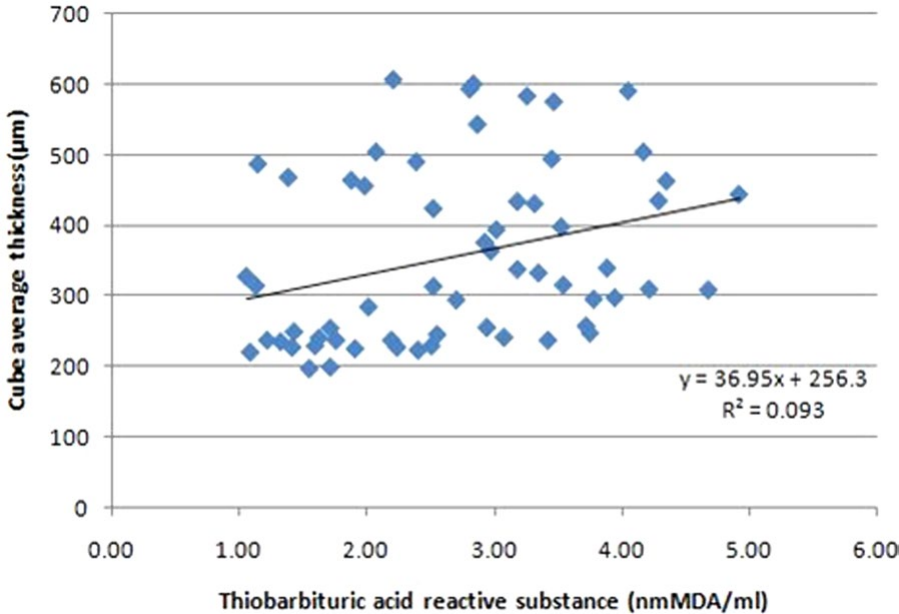
Scatter plot illustrating plasma levels of thiobarbituric acid reactive substances and cube average thickness among the study groups

**Fig. 5:**
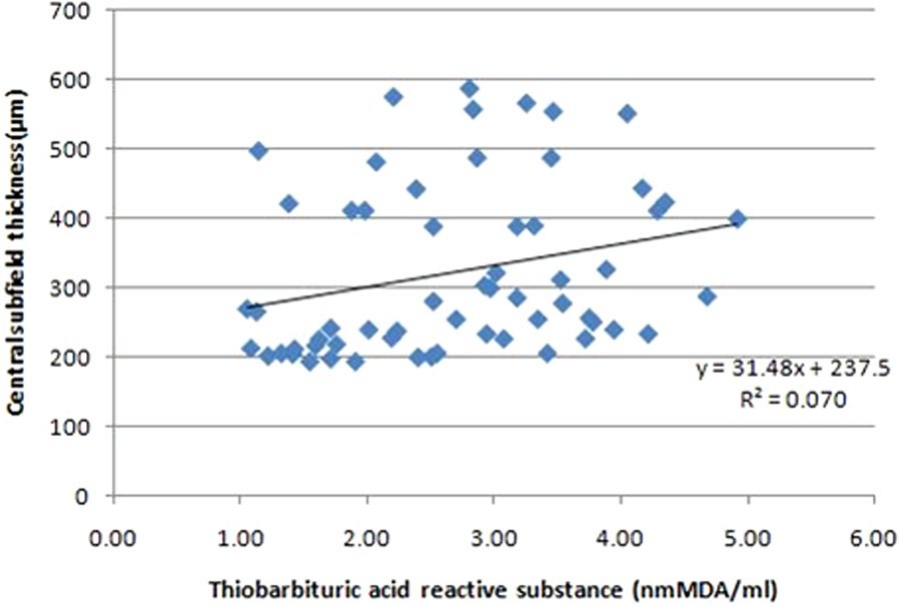
Scatter plot illustrating plasma levels of thiobarbituric acid reactive substances and central subfield thickness among the study groups

**Fig. 6:**
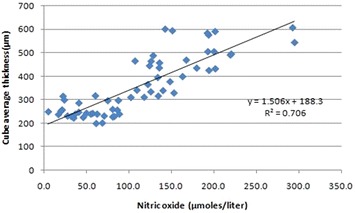
Scatter plot illustrating plasma levels of nitric oxide and cube average thickness among the study groups

**Fig. 7:**
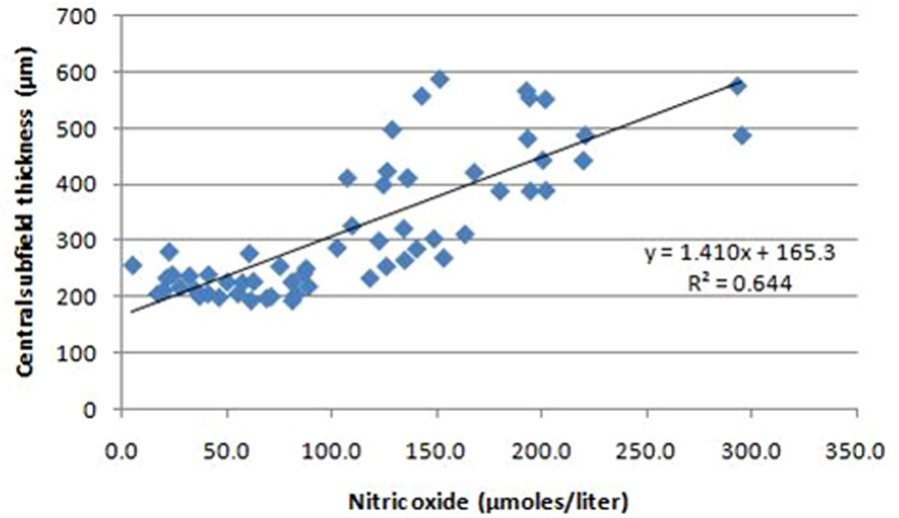
Scatter plot illustrating plasma levels of nitric oxide and central subfield thickness among the study groups

Pearson’s correlation analysis revealed a positive correlation of TBARS with CST (r = 0.29; p = 0.038) and CAT (r = 0.31; p = 0.04) (Figs. 4, 5). A positive correlation of NO with CST (r = 0.27; p = 0.03) and CAT (r = 0.7; p = 0.001) was also observed (Figs. 6, 7).

**Fig. 8:**
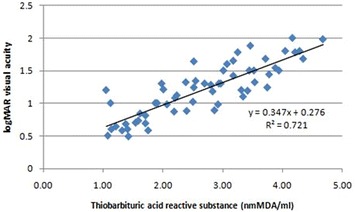
Scatter plot illustrating plasma levels of thiobarbituric acid reactive substances and logMAR visual acuity among the study groups

**Fig. 9:**
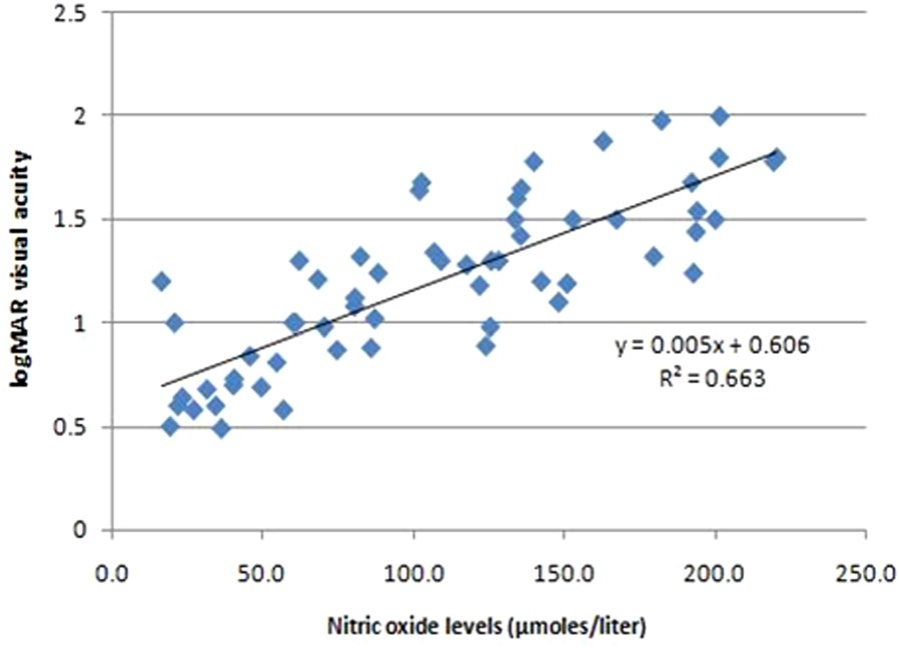
Scatter plot illustrating plasma levels of nitric oxide and logMAR visual acuity among the study groups

**Fig. 10:**
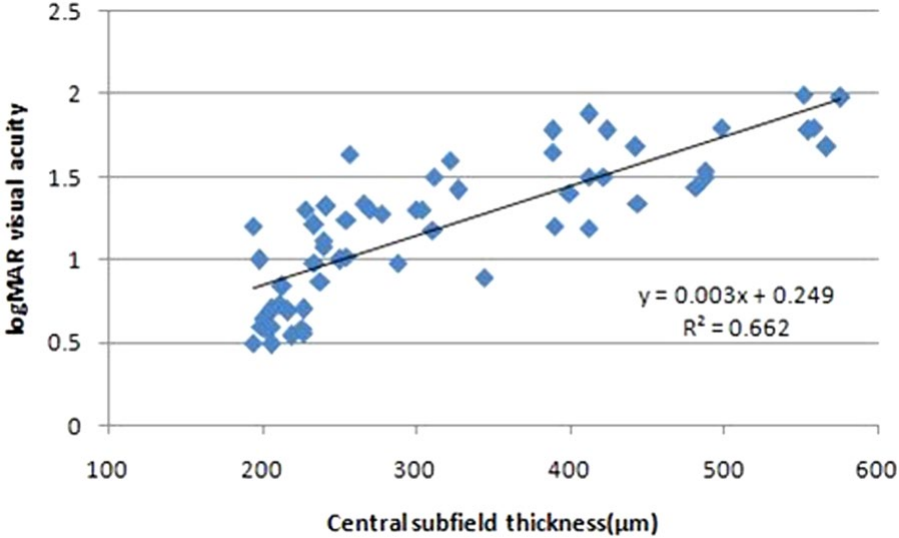
Scatter plot illustrating central subfield thickness and logMAR visual acuity among the study groups

On univariate analysis with logMAR visual acuity as dependent variable and TBARS, NO, CST, CAT as independent variables, a significant increase in visual acuity was observed with increase in TBARS (B = 0.22; p = 0.004), NO (B = 0.006; p < 0.001), CST (B = 0.005; p < 0.001) and CAT (B = 0.005; p < 0.001). On multivariate linear regression analysis with logMAR visual acuity as dependent variable and TBARS, NO, CST, CAT as independent variables and adjusting for other factors like duration of diabetes and HbA1c, it was observed that increase in TBARS (B = 0.07), NO (B = 0.001) and CST (B = 0.004) independently predict increase in logMAR visual acuity (p < 0.001) (Table 2) (Figs. 8, 9, 10).

**Table 2:** Univariate linear regression analysis taking visual acuity as a dependent variable with TBARS, NO, CST and CAT as independent variables followed by multivariate linear regression analysis adjusted for duration of diabetes and HbA1c

Independent variables	logMar visual acuity as dependent variable			
	Univariate analysis		Multivariate analysis (adjusted) (r^2^ = 0.96)	
	B	p	B	p
TBARS	0.22	0.004	0.07	<0.001
NO	0.006	<0.001	0.001	0.001
CST	0.005	<0.001	0.004	<0.001
CAT	0.005	<0.001	0.001	0.41

## Discussion

In the present study, a significant positive correlation of TBARS and nitric oxide levels in plasma, was observed with CST and CAT on imaging in diabetic retinopathy.

Significantly raised plasma levels of thiobarbituric acid reactive substances and metabolites of nitric oxide have been documented in patients of diabetic retinopathy [31, 32]. Izumi et al. found an increase in plasma levels of nitric oxide in patients with diabetes with or without DR in comparison to patients with no diabetes mellitus. Ozden et al. documented a significant increase in the serum levels of nitric oxide with increased severity of diabetic retinopathy [33]. Attenuation of oxidative and nitrative stress by anti-oxidant treatment in diabetic animals further corroborate these findings [34, 35]. Hartnett et al. published the possible role of oxidative stress markers including TBARS in predicting, diagnosing and preventing DR.

Endothelial damage due to accumulation of AGEs, activation of PKC, increased expression of VEGF and intracellular adhesion molecule (ICAM-1), increase in ROS leads to breakdown of BRB resulting in diabetic macular edema [36–39]. Studies on streptozocin induced diabetic rats have associated increased expression of endothelial NOS with BRB breakdown [40]. Our study found a positive correlation between the serum levels of TBARS, NO and CAT and CST on imaging. Subclinical macular edema identified by OCT has been suggested as potential organ-specific biomarkers of DR [27].

Retinal expression of VEGF is elevated by ROS [14]. Increased expression of VEGF has been documented to increase expression of NOS [40]. Formation of peroxynitrite due to reaction between ROS and nitric oxide further causes endothelial dysfunction [41]. Damage to the mitochondrial lipid membrane by ROS, result in increased apoptosis of retinal capillary cells [42]. Increase nitrative stress in the retinal vascular cells via activation of NF-*k*B by AGEs also lead to apoptosis of retinal pericytes [19]. Increased plasma levels of nitrotyrosine, another reactive nitrogen species, in type 2 diabetic patients were observed to have a significant positive correlations with plasma levels of ICAM-1 [43, 44]. In our previous study, increase in serum levels of VEGF and ICAM-1 with increase in severity of diabetic retinopathy was documented [45]. In a recent study, we documented an increase in structural alterations in outer retina with increase in oxidative and nitrative stress in diabetic retinopathy [46].

## Conclusion

In the present study, TBARS and NO were found to increase with the severity of retinopathy. TBARS and NO serve as potential biochemical biomarkers. Similar observations were obtained with CST and CAT. In a clinical retinal setting, CST and CAT serve as potential biomarkers for medical imaging for early recognition of increase in severity of diabetic retinopathy. Significant correlation between TBARS, NO and CST and CAT was recognized. TBARS, NO and CST would serve as significant indicators for prognosticating visual outcome in diabetic retinopathy.

## Abbreviations

TBARS: thiobarbituric acid reactive substance
NO: nitric oxide
CST: central subfield thickness
CAT: cube average thickness
SD-OCT: spectral domain optical coherence tomography
ETDRS: early treatment diabetic retinopathy study
NPDR: non proliferative diabetic retinopathy
PDR: proliferative diabetic retinopathy
BRB: blood retinal barrier
ROS: reactive oxygen species
AGE: advanced glycation end product
VEGF: vascular endothelial growth factor
PKC: protein kinase C
ICAM: intracellular adhesion molecule
